# Identification and validation of an immunological microenvironment signature and prediction model for epstein-barr virus positive lymphoma: Implications for immunotherapy

**DOI:** 10.3389/fonc.2022.970544

**Published:** 2022-09-29

**Authors:** Chenjiao Yao, Ruoyao Xu, Qianyuan Li, Sheng Xiao, Min Hu, Linyong Xu, Quan Zhuang

**Affiliations:** ^1^ Department of General medicine, The 3rd Xiangya Hospital, Central South University, Changsha, China; ^2^ Transplantation Center, The 3rd Xiangya Hospital, Central South University, Changsha, China; ^3^ Department of Pathology, The 3rd Xiangya Hospital, Central South University, Changsha, China; ^4^ Department of Hematology, The First Affiliated Hospital of Hainan Medical University, Haikou, China; ^5^ School of Life Science, Central South University, Changsha, China; ^6^ Research Center of National Health Ministry on Transplantation Medicine, Changsha, China

**Keywords:** EBV, lymphoma, CHIT1, TMEM163, macrophage

## Abstract

**Background:**

Epstein-Barr virus (EBV) is considered a carcinogenic virus, which is associated with high risk for poor prognosis in lymphoma patients, and there has been especially no satisfying and effective treatment for EBV+ lymphoma. We aimed to identify the immunological microenvironment molecular signatures which lead to the poor prognosis of EBV+ lymphoma patients.

**Methods:**

Differential genes were screened with microarray data from the GEO database (GSE38885, GSE34143 and GSE13996). The data of lymphoid neoplasm diffuse large B-cell lymphoma (DLBC) from the TCGA database and GSE4475 were used to identify the prognostic genes. The data of GSE38885, GSE34143, GSE132929, GSE58445 and GSE13996 were used to eluate the immune cell infiltration. Formalin-fixed, paraffin-embedded tissue was collected for Real Time Quantitative PCR from 30 clinical samples, including 15 EBV+ and 15 EBV- lymphoma patients.

**Results:**

Four differential genes between EBV+ and EBV- lymphoma patients were screened out with the significance of the survival and prognosis of lymphoma, including CHIT1, SIGLEC15, PLA2G2D and TMEM163. Using CIBERSORT to evaluate immune cell infiltration, we found the infiltration level of macrophages was significantly different between EBV+ and EBV- groups and was closely related to different genes. Preliminary clinical specimen verification identified that the expression levels of CHIT1 and TMEM163 were different between EBV+ and EBV- groups.

**Conclusions:**

Our data suggest that differences in expression levels of CHIT1 and TMEM163 and macrophage infiltration levels may be important drivers of poor prognosis of EBV+ lymphoma patients. These hub genes may provide new insights into the prognosis and therapeutic target for EBV+ lymphoma.

## Introduction

Epstein Barr virus (EBV), a human γ-1 herpesvirus, is associated with lymphoma pathogenesis, and over 90% of adults have antibodies to this virus (1). According to the World Health Organization (WHO), lymphoma is classified as non-Hodgkin’s (NHLs, including mature B-cell, mature T-cell, NK-cell types) and Hodgkin’s (2). EBV is related to multiple NHLs (natural killer/T-cell lymphoma (NKTCL) (3), follicular lymphoma (4), diffuse large B-cell lymphoma (5)) with poor prognosis. Besides NHLs, the adverse prognosis of Hodgkin Lymphoma (HL) with EBV infection is also statistically significant (6, 7). In general, EBV infection leads to poor prognosis of lymphoma patients. Although the immunotherapy techniques have developed rapidly in recent years, the therapies of EBV+ lymphoma is still based on radiotherapy and chemotherapy, combined with antiviral drugs (8).

Evidence of EBV associated malignancies suggests that the virus has the ability to actively shape the immune microenvironment (IME) (9). IME is increasingly recognized as a key factor in disease progression and therapeutic outcome, as well as playing a role in multiple stages of evading immune surveillance. There are a variety of infiltrating immune cells in the tumor IME (TIME), including T cells, B cells, macrophages, NK cells, myelogenic inhibitory cells and dendritic cells (10). Studies showed that the number of macrophages in IME was closely related to the prognosis of HL (11). At the same time, tumor associated macrophage (TAM) has a negative impact on the grade and survival of NHL (3, 12). Generally, a large number of M2 macrophages in TIME were associated with poor prognosis, while M1 macrophages were thought to be associated with good prognosis (11). However, new study shows that M1 macrophages exposed to apoptotic lymphoma cells displayed increased lymphoma growth-promoting activities (13). EBV infection also affects the macrophage behavior, which may be by promoting the expression of macrophage migration inhibitory factor (MIF) (14). In addition, MIF plays a leading role in the functional polarization of M2 macrophages, and MIF deficiency spontaneously restores M1 polarization (15). Therefore, EBV may affect the survival and prognosis of lymphoma patients by affecting macrophages in the IME.

Since the datasets of EBV+ and EBV- lymphoma tend to pay more attention to the differences in non-coding RNA but little attention to the differences in mRNA, preliminary studies were performed on GSE38885 (including posttransplant lymphoma). We identified the differential genes associated with poor prognosis of immunocompetent EBV+ lymphoma patients, and the expression levels of these meaningful differential genes will be measured in immunocompetent patients. Then we found significant changes in macrophage infiltration level by CIBERSORT, further exploring the relationship between differential genes and macrophages infiltration level. Our study intended to provide novel ideas for the treatment of lymphoma patients.

## Materials and methods

### Patient cohort and data preparation

The cohort of the study contained 8 datasets from the Gene Expression Omnibus (GEO, available at: https://www.ncbi.nlm.nih.gov/geo/) database and DLBC samples in TCGA (https://portal.gdc.cancer.gov/). The microarray data of GSE38885 included 44 lymphoma patients’ lymph nodes samples, 11 gut-associated lymphoid tissue samples and 10 other samples. In GSE38885 (31 EBV+ patients and 34 EBV-), 51 diffuse large B-cell lymphoma (DLBCL), 9 burkitt-like lymphoma and 5 burkitt lymphoma were included. Besides, there were 40 immunocompromised patients and 25 immunocompetent patients. In datasets we used, only GSE38885 included immunocompromised patients. The microarray data of GSE34143 included 3 EBV+ peripheral T cell lymphoma patients. The microarray data of GSE13996 included 18 EBV+ and 33 EBV- patients, which were lymph nodes samples from the tumor areas in Hodgkin’s lymphoma patients. GSE4475, GSE39133, GSE39134 and DLBC in TCGA included lymphoma patients’ survival data. 159 NHL patients from the GSE4475 included 9 aggressive B-NHL unclassifiable, 22 atypical BL, 5 BL and 123 DLBCL. 58 HLs patients GSE39133 and GSE39134, and 48 DLBC patients in TCGA included survival data. GSE132929 included 227 NHLs deleted germinal center B-cell-like diffuse large B-cell lymphoma (GC-DLBCL). GSE58445 included 147 peripheral T-cell lymphoma patients. Removing batch effects was performed using the Sangerbox tools by sva packages, a free online platform for data analysis (http://www.sangerbox.com/tool).

### Identification of prognostic differential genes

Patients were divided into two groups (EBV+/EBV-). The limma algorithm was applied to identify differential genes between EBV+ and EBV- groups. Genes with an FDR adjusted p-value <0.05 and an absolute value of log2 (fold change) >1 were considered as EBV-related differential genes. Based on median gene expression of differential genes, samples were then divided into high- and low-expression groups. To obtain EBV-related prognostic differential genes, univariate Cox analyses were further performed to screen all differential genes. Those with a p<0.05 were considered as significant.

### Identification of prognostic immune cells in immune microenvironment

Immune infiltration analysis based on 22 cell types identification by estimating relative subsets of known RNA transcripts (CIBERSORT) method. The GSE38885, GSE34143, GSE132929, GSE58445 and GSE13996 datasets were used as the gene expression input and LM22 (22 immune cell types) was set as the signature gene file. The analysis was conducted with 1,000 permutations. Patients were also divided into groups (EBV+/EBV- and immunocompromised/immunocompetent).

### Functional and pathway enrichment analysis

Functional enrichment of overlapping genes based on GO and KEGG annotations (top five modules) was performed using the Metascape tool (16). The obtained differential genes were subjected to multi-gene GO\KEGG enrichment analysis in Metascape Database (16). In DrugBank database (https://go.drugbank.com/) and PubChem database (https://pubchem.ncbi.nlm.nih.gov/), the above genes were used as targets to screen out related targeted drugs.

### Preliminary validation of clinical specimens

Archived formalin-fixed, paraffin-embedded (FFPE) tissue was retrieved from the Department of Pathology under the approval of the Institutional Review Board of Third Xiangya Hospital, Central South University (No.21155). All FFPE tissues with a histologically proven diagnosis of non-Hodgkin’s lymphoma were stored at 2 ~ 8°C within 3 years. The histologic diagnosis of all tissue specimens was made according to the 2016 WHO Classification of lymphoma (**Table 1**).

**Table 1:** Clinical characteristics and diagnosis information in 30 non-Hodgkin’s lymphoma patients.

Characteristic	EBV-positive	EBV-negative
Diagnosis, n	15	15
NK-T cell lymphoma	12	0
Burkitt lymphoma	2	0
Diffuse large B-cell lymphoma	1	8
Peripheral T-cell lymphoma	0	3
Mantle cell lymphoma	0	1
Chronic lymphocytic leukemia/small lymphocytic lymphoma	0	1
Enteropathy-associated T-cell lymphoma	0	1
Plasmablastic lymphoma	0	1
Sex, n	15	15
Male	8	9
Female	7	6

### RNA extraction and quantitative PCR

Real Time Quantitative PCR (RT-qPCR) was used to quantitative expression of prognostic differential genes. Total RNA was extracted using the FFPE RNA Kit (AmoyDx, Xiamen, China) following the manufacturer’s protocols and was quantified using a NanoDrop 2000 spectrophotometer (Thermo Fisher Scientific, USA). Templates were amplified using HiScript II U+ One Step RT-qPCR Probe Kit (Vazyme Cat#Q222-CN, Nanjing, China) on a LightCycler 480 II Real-Time PCR System. Primers and probes were designed applying IDT online software (sg.idtdna.com/PrimerQuest/) and were synthesized by Sangon Biotech (Shanghai, China).

The thermal cycler conditions comprised as followed: reverse transcription 55°C for 15 min, predenaturation 95°C for 30 s, 45 cycles of denaturation at 95°C for 10 s, annealing 60°C for 30 s, and extension at 72°C for 30 s. The gene expression level was normalized using the endogenous control gene ribosomal protein L13a (RPL13A) (**Table 2**).

**Table 2:** Primer sequences of RPL13A, CHIT1, SIGLEC15, PLA2G2D and TMEM163.

Gene	Primer-probe
RPL13A	Forward 5’-ATCCACTACCGGAAGAAGAAAC-’3 (Sense)
	Probe with 5’-6-FAM 5’-TCCACGTTCTTCTCGGCCTGTTTC-’3 (Anti-Sense)
	Reverse 5’- GTCTTGAGGACCTCTGTGTATTT-’3 (Anti-Sense)
CHIT1	Forward 5’-GGCACTCAGAAGTTCACAGATA-’3 (Sense)
	Probe with 5’-VIC 5’-TGTCAACTCGGCCATCAGGTTTCT-’3 (Sense)
	Reverse 5’-AGGCCGTCAAAGCTGTATT-’3 (Anti-Sense)
SIGLEC15	Forward 5’-CCTCCTATGTGGACAACCATTT-’3 (Sense)
	Probe with 5’-CY5 5’-ACGTCTGTGTGTGTGTGTGTGTGT-’3 (Sense)
	Reverse 5’-CGTGTACTCTCTCTCTCTCTCTC-’3 (Anti-Sense)
PLA2G2D	Forward 5’-CTGACTGAGCAGTTCTGAGATG-’3 (Sense)
	Probe with 5’-VIC 5’-ATAGGAATGAAGGACTGTGGGCGC-’3 (Sense)
	Reverse 5’-TACCTGGAGTGGTGCATAGA-’3 (Anti-Sense)
TMEM163	Forward 5’-GCCCAGAACTACAGGAAGAAG -’3(Sense)
	Probe with 5’-CY5 5’- CATTGTGGGTGTCCTGGTTCTCCA-’3 (Sense)
	Reverse 5’- CCTCATAACGGAGACAGTAAAGG-’3 (Anti-Sense)

### Immunohistochemistry

The FFPE tissues were deparaffinized and boiled in citrate buffer (MXB Cat#MVS-0100, Fuzhou, China) for antigen retrieval. Endogenous peroxidase was blocked by 3% H2O2. Then, slides were blocked in serum, incubated with anti-PLA2G2D antibody (diluted 1:50, Ancepta Cat# AP11151b) or anti-TMEM163 antibody (diluted 1:50, Invitrogen Cat# PA5-114329) at 37°C for 1h, incubated with an anti-rabbit secondary antibody, and visualized with diaminobenzidine (MXB Cat# DAB-2031, Fuzhou, China). The images of IHC staining were captured with a microscope.

### Statistical analysis

The RT-qPCR results were repeated at least three times in independent experiments and data are expressed as means with SEM or 95% CIs. Statistical comparisons were performed with unpaired t test with Welch’s correction. Statistical analysis was carried out using Graphpad prism version 9.1.1 (GraphPad Software Inc., La Jolla, CA, USA). All tests were two tailed, and a P<0.05 was considered statistically significant. The limma and cibersort algorithms were performed in R version 4.0.5 (https://www.r-project.org/).

**Figure 1:**
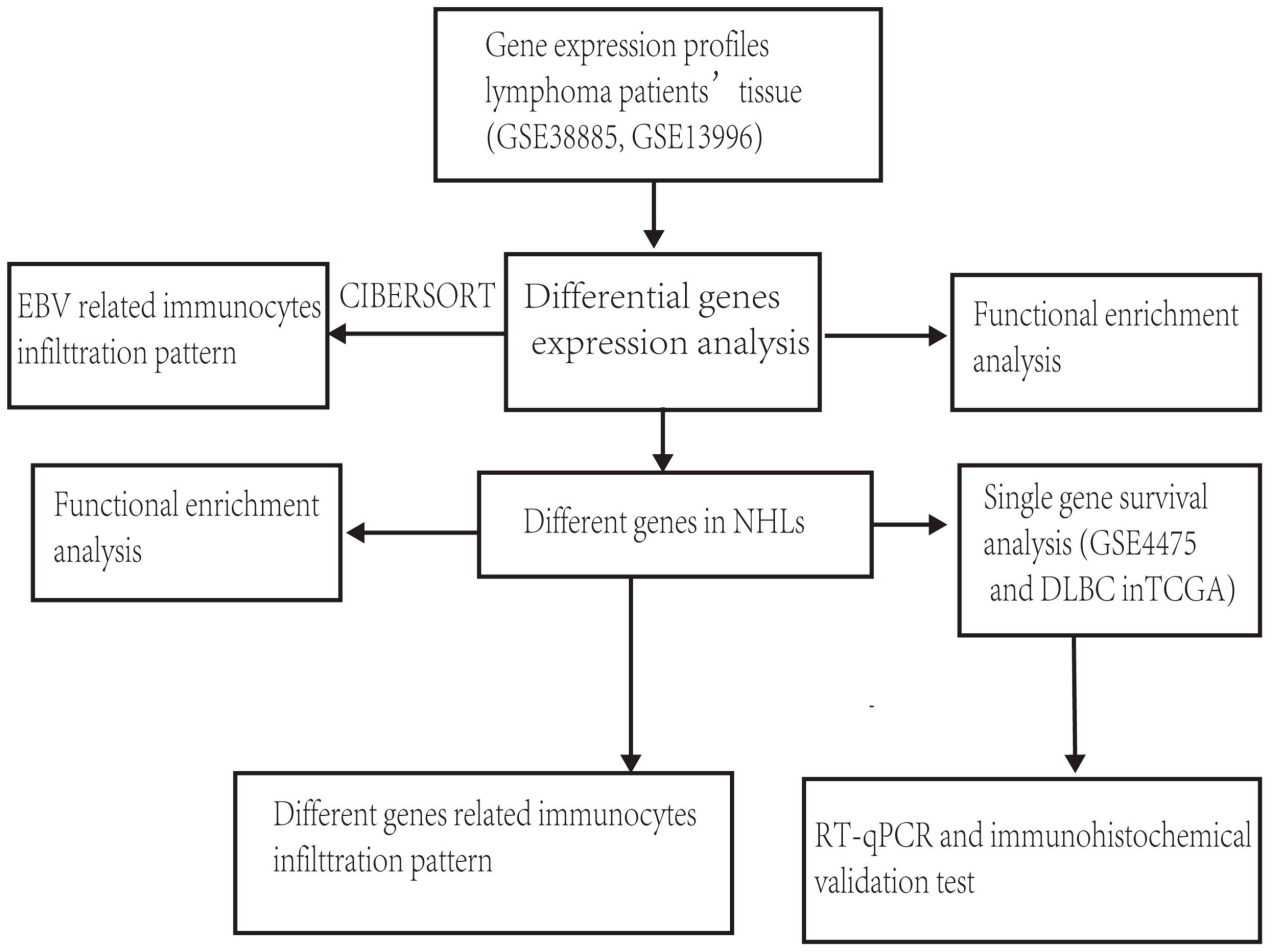
Flow chart of methodologies is used in this study.

The general idea and methodologies used in this study were drawn as a flow chart (**Figure 1**).

## Results

### Differential genes are associated with the prognosis of EBV+ lymphoma

**Figure 2:**
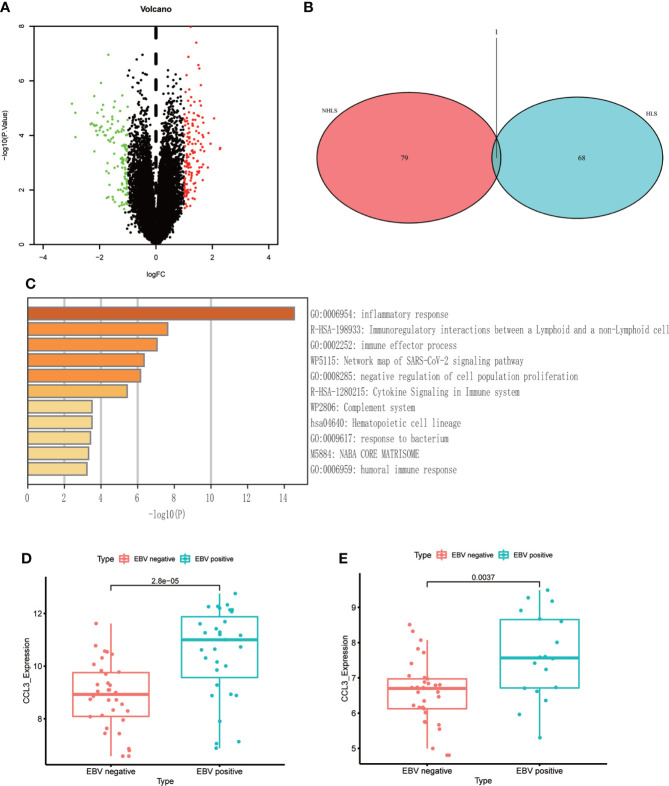
The differential genes between EBV+ and EBV- lymphoma samples. **(A)** Volcano plot of differential genes in EBV- and EBV+ lymphoma groups. **(B)** Venn diagram of overlapping differentially expressed genes in GSE38885 and GSE13996 datasets. **(C)** Functional enrichment of differential genes bases on Metascape database. **(D)** The box plot shows the expression level of CCL3 between two groups in NHLs. **(E)** The box plot shows the expression level of CCL3 between two groups in HLs.

116 patients were divided into EBV+ and EBV- groups (GSE38885, GSE13996). Compared to EBV- group, 21 up-regulated genes and 14 down-regulated genes were obtained (|logFC|>1; P<0.05) in the EBV+ group (**Figure 2A**). Results of functional enrichment analysis showed that enrichment in genes involved in inflammatory response, immune response and immune cells, suggesting that EBV may lead to poor prognosis of patients by affecting the immune system (**Figure 2C**). After NHL and HL sample data were integrated into EBV+ and EBV-groups, only a few genes (35 genes) could be obtained after differential analysis. We speculate that EBV may affect NHLs and HLs differently. Since there are significant differences between NHLs and HLs in pathological manifestations, degree of malignancy and survival prognosis, we identified the effects of EBV on the gene expression levels of NHLs and HLs respectively. We identified only one overlapping gene (CCL3) in NHLs and HLs cohorts *via* a Venn diagram (**Figures 2B, D, E**). Because GSE38885 included immunocompetent lymphomas and post-transplant lymphomas which were immunocompromised lymphoma patients, we isolated them to observe the expression level of CCL3. Only a small number of immunocompetent EBV+/- lymphomas could be obtained in GEO, differential analysis could not be carried out. However, high expression of CCL3 was also found in EBV+ immunocompromised lymphomas (30 EBV+ and 10 EBV-) (**Supplementary Figure S1**).

### CCL3 suggests that EBV mainly affects macrophages in the immune microenvironment

**Figure 3:**
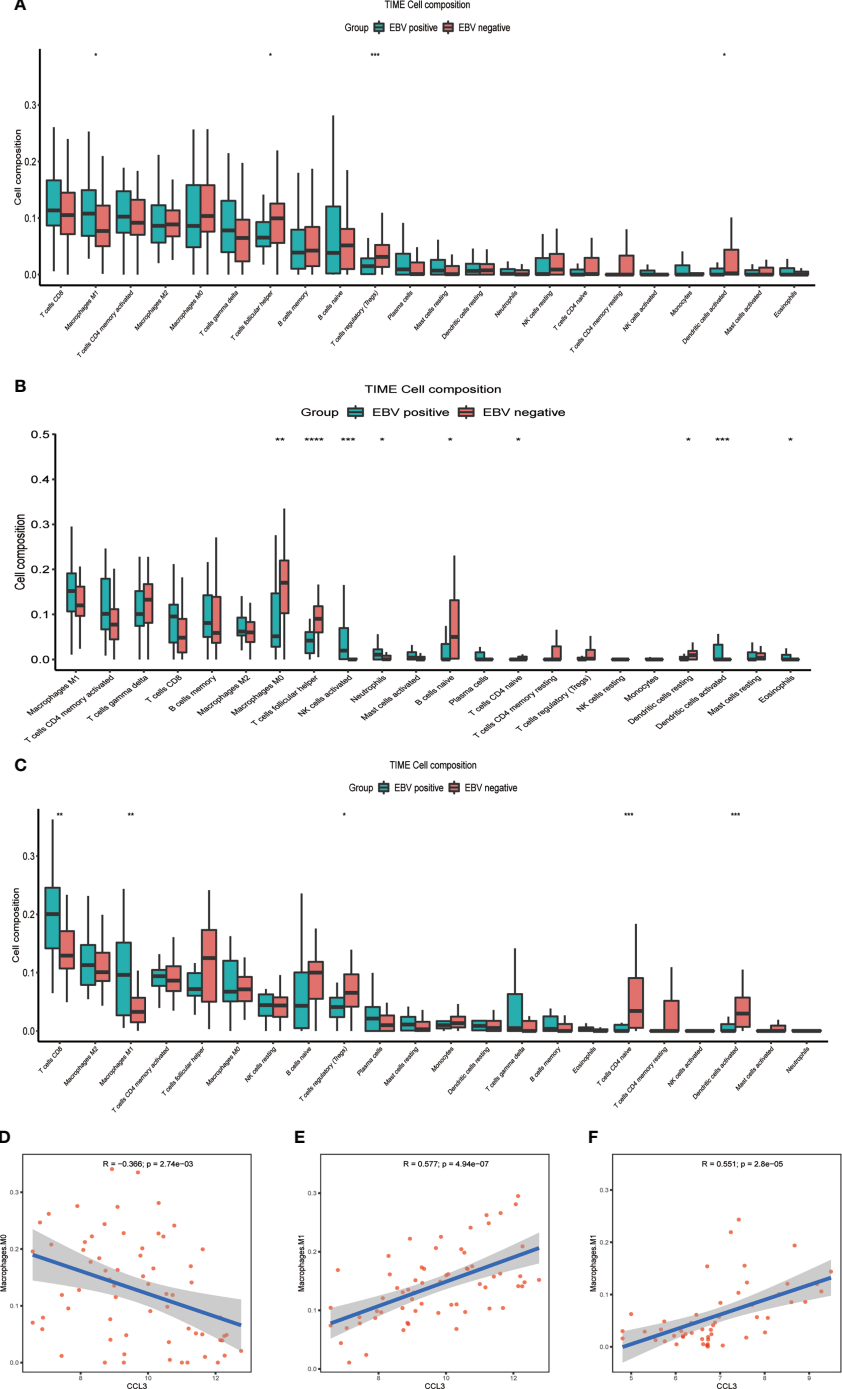
The difference between immune cell fractions between EBV+ and EBV- group. **(A)** The box plot shows the differences of infiltration of 22 kinds of immune cells between two groups in lymphoma samples. **(B)** The box plot shows the differences of infiltration of 22 kinds of immune cells between two groups in NHLs. **(C)** The box plot shows the differences of infiltration of 22 kinds of immune cells between two groups in HLs. **(D, E)** Correlation of CCL3 with infiltration of macrophage in NHLs. **(F)** Correlation of CCL3 with infiltration of macrophage in HLs *<0.05, **<0.01, ***<0.001, ****<0.0001.

CCL3 also known as macrophage inflammatory protein 1 alpha, which has the chemotactic action on macrophages to inflammatory sites (17). In our data, CCL3 is the only differential gene that exists in both NHLs and HLs, and a chemokine of macrophages, which is an important component of immune microenvironment. Based on the above two points, we speculated that changes in expression level and immune microenvironment jointly affect the prognosis of EBV+ lymphoma patients. Further, we evaluated the landscape of IME cell infiltration models systematically by CIBERSORT algorithm. In lymphoma samples (GSE38885 and GSE13996), we found that macrophages M1, T cell follicular helper, and T cells regulatory (Tregs) were significantly changed between EBV+ and EBV- group (**Figure 3A**). **Figures 3B, C** summarize the findings obtained from NHLs and HLs samples. Obviously, the proportions of TIME cells in NHLs and HLs varies significantly between both intra- and intergroup. The fraction of total T cells and total macrophages were higher in TIME cells than other cells, and T cells and macrophages were significantly changed between EBV+ and EBV- group in NHLs and HLs samples. CCL3 was negatively correlated with M0 macrophages, but positively correlated with M1 macrophages in 65 NHLs from GSE38885 (**Figures 3D, E**), while CCL3 was positively correlated with M1 macrophages in 51 HLs from GSE13996 (**Figure 3F**). Besides, CCL3 was also positively correlated with M1 macrophages in immunocompetent NHLs (GSE132929) (**Supplementary Figure S2**). Combined with the results of the new study and our analysis, we speculated that M1 macrophages is a protumor activation state in lymphoma. EBV may have different effects on macrophages in different types of lymphoma environments, so analysis should focus on NHLs or HLs.

### EBV affects the prognosis of NHLs patients

**Figure 4:**
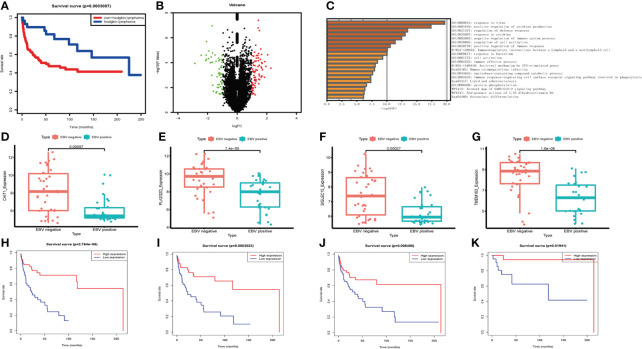
The expression levels of differential genes influencing the survival of NHL patients. **(A)** Survival analysis of lymphoma patients between NHLs and HLs. **(B)** Volcano plot of differential genes in EBV- and EBV+ lymphoma group. **(C)** Functional enrichment of differential genes bases on Metascape database. **(D–G)** The box plot shows the expression levels of four differential genes between two groups in NHLs. **(H–K)** Correlation of differential gene expression level with overall survival.

By analyzing the survival data of three datasets GSE4475 (159 NHLs), GSE39133 and GSE39134 (58 HLs), it was found that NHLs patients had a worse prognosis than HLs (**Figure 4A**). Therefore, we focus on the influence of EBV on NHLs. 65 patients (GSE38885) were divided into EBV+ and EBV- groups. Compared to EBV- group, 79 genes were up-regulated and 72 genes were down-regulated in the EBV+ group (**Figure 4B**). 151 differential genes, 79 up-regulated genes and 72 down-regulated genes were used in function enrichment analysis, respectively. After the analysis, response to virus, regulation of immune system and immune cells were found in all enrichment results (**Figure 4C**; **Supplementary Figures S3A, B**). Further survival analysis showed four down-regulated genes (**Figures 4D–G**) were strongly associated with poor prognosis of NHLs patients. High expression of CHIT1, SIGLEC15, PLA2G2D, and TMEM163 predicted longer survival in the NHLs from TCGA-DLBC and GSE4475 without germinal center B-cell-like lymphoma (**Figures 4H–K**). Combined with the results of functional enrichment, it is speculated that these four genes may improve the prognosis of patients by influencing IME.

### The effect of EBV on macrophage infiltration level in NHLs

**Figure 5:**
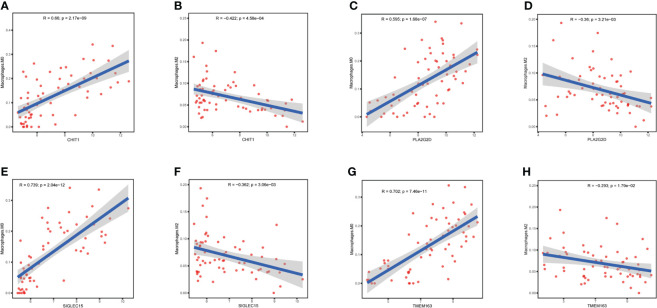
Correlation of differential genes with infiltration of macrophage in NHL patients **(A, B)** Correlation of CHIT1 with infiltration of macrophage M0 and M2 in NHL patients. **(C, D)** Correlation of PLA2G2D with infiltration of macrophage M0 and M2 in NHL patients. **(E, F)** Correlation of SIGLEC15 with infiltration of macrophage M0 and M2 in NHL patients. **(G, H)** Correlation of TMEM163 with infiltration of macrophage M0 and M2 in NHL patients..

We further investigated whether CHIT1, SIGLEC15, PLA2G2D, and TMEM163 affected the immune microenvironment in NHLs patients (GSE38885). CIBERSORT was used to estimate the infiltration of 22 kinds of immune cells in the samples (GSE38885) and the infiltration level of macrophages was the highest, among which the infiltration level of M0 macrophages was significantly different in the EBV+ and EBV- groups, indicating that macrophages should be the main component in the immune microenvironment of lymphoma patients. Meanwhile, the infiltration level of M0 macrophages in the EBV- group was significantly higher than that in the EBV+ group, suggesting that M0 macrophages may be a key factor affecting the prognosis of lymphoma patients (**Figure 3A**). Correlation analysis showed that CHIT1, SIGLEC15, PLA2G2D, and TMEM163 were significantly correlated with the infiltration level of macrophage. It was positively correlated with M0 macrophage infiltration (**Figures 5A, C, E, G**) and negatively correlated with M2 macrophage infiltration (**Figures 5B, D, F, H**). The role of M0, M2 macrophages in tumor immune microenvironment is consistent with the favorable genes of CHIT1, SIGLEC15, PLA2G2D and TMEM163 for the prognosis of lymphoma patients. At the same time, we also analyze correlation between four genes and macrophage infiltration in immunocompetent NHLs (GSE132929 and GSE58445), and the results were basically consistent with GSE38885 (**Supplementary Figure S4**). Therefore, CHIT1, SIGLEC15, PLA2G2D, and TMEM163 may influence the prognosis of patients by influencing the infiltration level of macrophages in immune cells.

### Preliminary validation of clinical specimens

**Figure 6:**
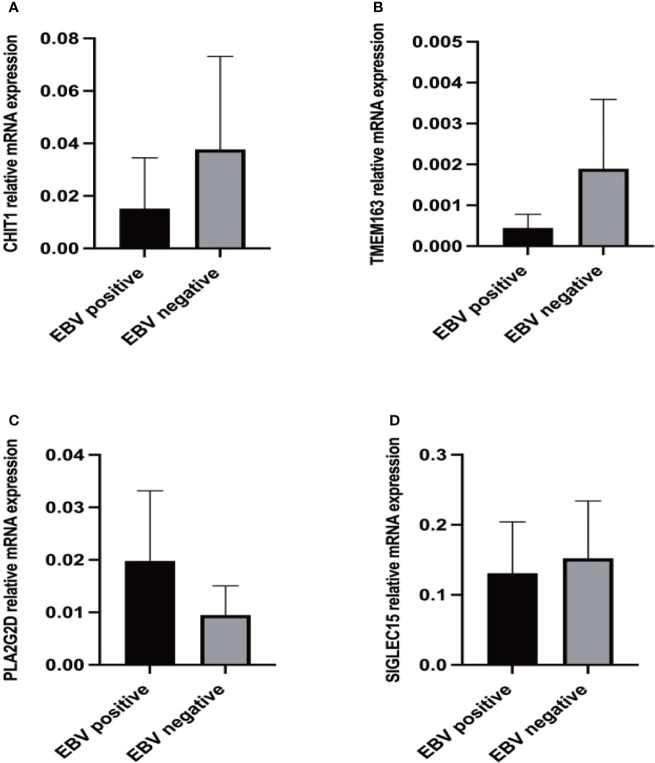
Expression levels of differential genes in NHL patients. **(A)** RT-qPCR results of CHIT1 in clinical samples. **(B)** RT-qPCR results of TMEM163 in clinical samples. **(C)** RT-qPCR results of PLA2G2D in clinical samples. **(D)** RT-qPCR results of SIGLEC15 in clinical samples.

We further validated CHIT1, SIGLEC15, PLA2G2D and TMEM163 mRNA expression levels in 30 NHLs patients using RT-qPCR. The mRNA expressions of CHIT1 and TMEM163 were lower in 15 EBV+ NHLs patients compared with 15 EBV- NHLs patients as we expected (**Figures 6A, B**), but there was no significant difference between the two groups. And the mRNA expression of PLA2G2D seems to be higher in 15 EBV- NHLs patients compared with 15 EBV+ NHLs patients even though the difference was not significant, either (**Figure 6C**). There was a rising trend of SIGLEC15 mRNA in the EBV- group, but not a significant difference (**Figure 6D**). The similar results were obtained when GC-DLBCL was deleted from the samples (13 EBV+ and 13 EBV-) (**Supplementary Figure S5**).

### Targeted drugs of hub genes at Drugbank and PubChem

These genes are associated with poor prognosis of EBV+ lymphoma patients, and the results of immune infiltration analysis showed that the expression level of these genes is associated with macrophages in the immune microenvironment, so increasing the expression level of these genes will be helpful for the therapy of EBV+ NHL. The above-mentioned genes can be used as targets in DrugBank and PubChem to find corresponding targeted drugs. The targeted drugs of CHIT1 and PLA2G2D are obtained from Drugbank, and the targeted drugs of SIGLEC15 are obtained from PubChem.

## Discussion

According to the cancer statistics in 2013, the incidence of lymphoma was 4.2 per 100,000 and the mortality was 2.2 per 100,000 in mainland China (18). Some recent breakthroughs in immunotherapy have provided a new strategy for effective oncotherapy. However, the differences in gene expression levels between EBV+ and EBV- NHL have received little attention. There is a lack of sufficient datasets for RNA-seq of EBV+/- NHL patients’ tissues in the GEO database. Therefore, we could only use the dataset including immunocompromised lymphoma for differential analysis to help us find reliable hub genes to improve the prognosis of EBV+ NHLs patients and provide a personalized therapy for them. However, due to the small number of post-transplantation lymphoma patients, it is difficult to conduct experimental verification, so we integrated immunocompetent NHL patients in GSE38885 and GSE34143 (4 EBV+ and 18 EBV-). In immunocompetent NHLs, the average expression levels of the four genes in the EBV+ group were lower than those in the EBV- group (**Supplementary Figure S7**). We further verified the four genes in clinical samples. In addition, the difference in the average infiltration level of macrophage between the EBV+ and EBV- groups and the correlation of macrophage infiltration with the four differential genes were consistent with the results obtained in GSE38885 (**Supplementary Figure S8**).

Since immunocompromised lymphoma patients were involved in GSE38885, we further analyzed the differential genes that might affect the prognosis of different immune status lymphoma patients. We respectively analyzed in samples from NHL patients (GSE38885, TCGA-DLBC and GSE58445) and lymphoma patients containing HLs (GSE38885 and GSE13996), but only a small number of differential genes were obtained between immunocompetent and immunocompromised patients. After functional enrichment of differential genes (logFC>0.6), the results also showed the association of immune cells (**Supplementary Figure S9**). Besides, there was a trend that the average overall survival was worse in immunocompromised NHLs (GSE38885, TCGA-DLBC and GSE58445), although not statistically significant (**Supplementary Figure S10B**). CHIT1 was the only gene that was associated with overall survival (**Supplementary Figure S10C**). Besides, CHIT1 was also associated with the expression levels of T cells CD4 memory activated, T cells follicular helper, eosinophils and dendritic cells activated, which were significantly different in NHLs with different immune status (**Supplementary Figures S10D–H**). In the analysis of whole lymphoma samples (GSE38885 and GSE13996), we also found only a small number of differential genes (logFC>0.8) (**Supplementary Figure S11A**). Due to the lack of survival information in the datasets, we directly conducted immune infiltration analysis. These differential genes were correlated with the infiltration level of T cells CD8, T cells follicular helper and eosinophils, which were significantly different in lymphoma with different immune status (**Supplementary Figures S11B, C**). In previous studies, little attention has been paid to the differences between lymphomas with different immune status, and our study has only obtained a few differential genes. Therefore, it would be of more interest to analyze the differences between EBV+ and EBV- lymphoma. However, CHIT1 also showed differences in lymphoma patients with different immune status, affected the prognosis of lymphoma patients, and was associated with the infiltration of immune cells. In further studies, we will further study the effect of CHIT1 on lymphoma with different immune status.

When collecting clinical samples for validation, we found that the EBV+NHL patients were mainly NKT patients, and other types of NHL patients were less. However, NKT patients were less in EBV- NHL patients, and other types of NHL patients were the main types. Therefore, the clinical samples we collected were consistent with the proportion of each type NHL patients.

Here, we found that EBV mainly affects macrophages in IME of lymphoma patients through the differential gene CCL3. Although EBV infection may lead to poor prognosis of NHLs and HLs by different pathways, its effect on macrophages in the IME could be considered as one of the factors contributing to poor prognosis. We reported that down-regulation of CHIT1, SIGLEC15, PLA2G2D and TMEM163 was associated with poor prognosis in immunocompetent NHL, and expression levels of four genes were lower in lymphoma than in normal tissues. The down-regulation of CHIT1 and TMEM163 has been validated in the EBV+ immunocompetent NHLs group using RT-qPCR. Our results demonstrated that macrophages might play a role in EBV+ lymphoma and M0 macrophages are the most critical factor in EBV+ NHLs. Both in immunocompetent and immunocompromised NHLs, hub genes are associated with M0, M2 macrophage infiltration. Several articles have reported the role of macrophages in the immune microenvironment in lymphoma (13, 19, 20). M2 macrophages play a dominant role in the long-term IME, which is closely related to the growth, invasion and metastasis of tumor (21, 22). Contrary to our previous understanding of M1 macrophages, it plays a protumor role in lymphoma (13).The abundance ratio of M0 macrophages was positively correlated with prognosis (20). The result of CIBERSORT showed that four differential genes were positively correlated with M0 macrophage infiltration, but negatively correlated with M2 macrophage infiltration.

M0 is a dormant macrophage that can be polarized into functional M1 and M2 macrophages (23) and M1, M2 macrophages are unfavorable factors for the prognosis of lymphoma patients. The above hub genes may promote M0 macrophage infiltration, and inhibit the polarization of M0 into M1, M2 macrophages, thus promoting a good prognosis of the EBV+ lymphoma patients. All these results supported that down-regulation of these genes was related to poor prognosis of EBV+ lymphoma patients.

In PCR experiments results, we found that the expression level of the CHIT1 gene in EBV+ group is lower than EBV- group. Two deaths of EBV+ patients were observed in lymphoma patients included in the study, and in their PCR results we found that the CHIT1 gene in both patients had the significantly lowest expression in all samples. Therefore, the low expression of CHIT1 in EBV+ lymphoma patients was closely related to the poor prognosis and even death of patients. This may be related to the effect of the gene on macrophages. CHIT1 is widely considered as a marker to activate macrophages, which is mainly expressed in macrophages but not in tumor cells. At the same time, some studies have pointed out that it also has a certain regulatory significance in the process of complete maturation and polarization of macrophages (24). During mononuclear macrophage differentiation, protein synthesis and mRNA analysis showed that CHIT1 was significantly upregulated in M1 and M2 macrophages (25). The change of CHIT1 expression level was significantly correlated with the polarization of macrophages, and the expression level was different in M1 and M2 macrophages, suggesting that the expression level can influence the direction of polarization of macrophages (26). However, in our study, CHIT1 expression was positively correlated with M0 macrophage infiltration and negatively correlated with M2 macrophage infiltration. It may be that the immune microenvironment of lymphoma affects the regulation of CHIT1 on macrophages, which is worthy of further investigation.

There was no significant difference in SIGLEC15 gene expression between EBV+ and EBV- patients in RT-qPCR results. The expression of SIGLEC15 in two deceased was found to be the lowest in all samples. The gene showed a correlation with M0 macrophage infiltration in previous analyses. It has been linked to the prognosis of many cancers, which is a new tumor therapeutic target. The upregulation of SIGLEC15 was linked to longer overall survival in bladder urothelial carcinoma, breast invasive carcinoma, head and neck squamous cell carcinoma, thyroid carcinoma, and uterine corpus endometrial carcinoma, and longer relapse-free survival in breast invasive carcinoma, liver hepatocellular carcinoma, ovarian serous cystadenocarcinoma, and uterine corpus endometrial carcinoma (27). That article did not mention the relationship between the expression level of SIGLEC15 and the survival of lymphoma patients. Through our survival analysis and experiments, we found that SIGLEC15 is lowly expressed in EBV+ lymphoma patients and can lead to poor prognosis or even death in patients. SIGLEC15 could be used as a new therapeutic target to improve prognosis by upregulating expression level of SIGLEC15 in EBV+ patients.

The RT-qPCR results of TMEM163 agreed with our prediction. TMEM163 affects the infiltration of M0 and M2 macrophages, which may be related to the expression of a zinc ion transport protein. The protein can bind zinc and other divalent cations and recruit of them into cystic organelles. Meanwhile, zinc ions play an important role in macrophages and innate immunity. Zinc ion movement in and out of cells depends on the metal ion transport protein family SLC39, which is primarily responsible for the transport of ions from extracellular site of organelle to cytoplasm. On the other hand, the SLC30 family is primarily responsible for the transport of ions from the cytoplasm to the extracellular space or organelle. TMEM163 gene is a new zinc transporter with similar functions to SLC30 (28). Besides, studies have shown that the lack of SLC39A10, a member of the SLC39 family of metal ion transporters, can cause macrophages to be deficient in zinc ions, improving stability of p53 protein, which induces the apoptosis of macrophages (29). Therefore, we speculated that TMEM163 also regulated different subtypes of macrophages by affecting the transport of zinc ions or induce the death of certain subtypes of macrophages (such as M2 macrophage).

The expression of the PLA2G2D gene in the EBV+ group was higher than that in the EBV- group in both PCR and immunohistochemistry experiments, which was inconsistent with our previous analysis. However, high expression of PLA2G2D exists in lymphatic tissue itself, and the difference in expression between EBV+ and EBV- lymphoma patients may be masked by PLA2G2D expressed in tissue itself. In the process of experimental verification, it has a great influence on the experimental results, leading to the inconsistency between the experimental results and the analysis results.

In this study, although the dataset for the initial differential analysis included samples from immunocompromised lymphomas, the four hub genes obtained from the dataset were validated using immunocompetent NHL clinical samples, and both survival and immune infiltration analyses for the four hub genes were performed in immunocompetent NHL. Therefore, these four hub genes are still of general significance in NHL patients. Four genes were identified to affect the prognosis of lymphoma patients by influencing macrophages in the immune microenvironment. Higher proportions of M0 macrophages in IME predicted a better prognosis in lymphoma patients, while high proportions of M1 and M2 macrophages in IME predicted a worse prognosis. The functions of the targeted drugs mentioned above (**Table 3**) are still unclear, so the specific functions need to be further studied in order to obtain targeted drugs that can be applied clinical use. However, our study still suffers from some limitations. Due to the different incidence of different lymphoma types in the population, the number of patients with different types of EBV+ lymphoma in our clinical sample also varied. Besides, we also face the problem of insufficient sample size. But the preliminary clinical specimen validation suggested the reliability of previous conclusions, it can provide a novel idea for our clinical treatment. In the following studies, we will use single-cell sequencing and other methods to identify specific macrophage subgroup.

**Table 3:** Targeted drugs of related genes in Drugbank and PubChem.

TARGET	DRUGGBANK ID/PubChem CID	Drugs/substrates
Chitinase 1	DB03109DB03539	2-acetylamino-2-deoxy-b-D-allopyranose2-(Acetylamino)-2-Deoxy-6-O-Methyl-Alpha-D-Allopyranose
phospholipase A2gropu IID	DB03193DB04404	Stearic acid Lauric acid
sialic acid-binding Ig-like lectin 15	155553698	C40H67N5O27(real name is in supplementary material 2)
	155518853	C_42_H_70_N_6_O_27_ (real name is in supplementary material 2)

## Conclusions

We found that high hub genes expression is associated with favorable prognosis and increased immune cell infiltration in lymphoma, especially macrophages. Therefore, hub genes likely played a role in macrophages in the immune microenvironment. CHIT1 and TMEM163 have been identified using RT-qPCR and are able to function as potential novel therapeutic targets for EBV+ lymphoma patients.

## Data availability statement

The datasets presented in this study can be found in online repositories. The names of the repository/repositories and accession number(s) can be found in the article/**Supplementary Material**.

## Ethics statement

The studies involving human participants were reviewed and approved by The Institutional Review Board of Third Xiangya Hospital, Central South University (No.21155). The patients/participants provided their written informed consent to participate in this study.

## Author contributions

The study was conceived and designed by QZ. Statistical analyses were performed by CY and RX. Software and code were prepared by RX, MH and LX. Manuscript was written and revised by CY, RX, and QL. Validation experiments and specimen collection were performed by QL and SX. All authors contributed to this article and approved the submission. CY and RX are the co-first authors.

## Funding

This study was supported by grants from the National Natural Science Foundation of China (81700658) and (82270795), the Hunan Provincial Natural Science Foundation (2020JJ3058, 2021JJ30910), and the Key Research and Development program of Hainan Province (ZDYF2020135)

## Conflict of interest

The authors declare that the research was conducted in the absence of any commercial or financial relationships that could be construed as a potential conflict of interest.

## Publisher’s note

All claims expressed in this article are solely those of the authors and do not necessarily represent those of their affiliated organizations, or those of the publisher, the editors and the reviewers. Any product that may be evaluated in this article, or claim that may be made by its manufacturer, is not guaranteed or endorsed by the publisher.
