# Supplementary material for: Identification and validation of an immunological microenvironment signature and prediction model for epstein-barr virus positive lymphoma: Implications for immunotherapy
Source: Front Oncol. 2022 Sep 29;12:970544. doi: 10.3389/fonc.2022.970544 (PMC9559214; doi:10.3389/fonc.2022.970544)
Supplement: Supplementary file 1 [file DataSheet_1.pdf]

Supplementary figures and table explanation

1. Supplementary Figures

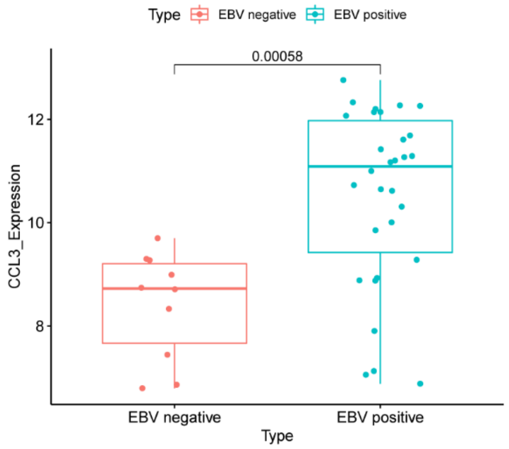

Supplementary Figure 1. The box plot shows the expression levels of CCL3 between two groups in immunocompromised NHLs from GSE38885 (30 EBV+ and 10 EBV-).

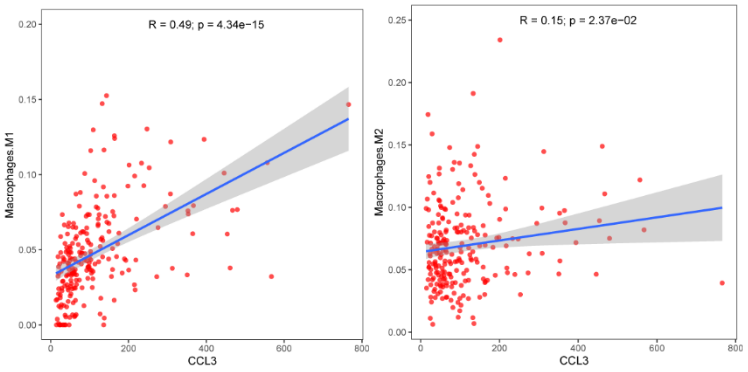

Supplementary Figure 2. Correlation of CCL3 with infiltration of macrophage in NHLs without GC-DLBC (GSE132929).

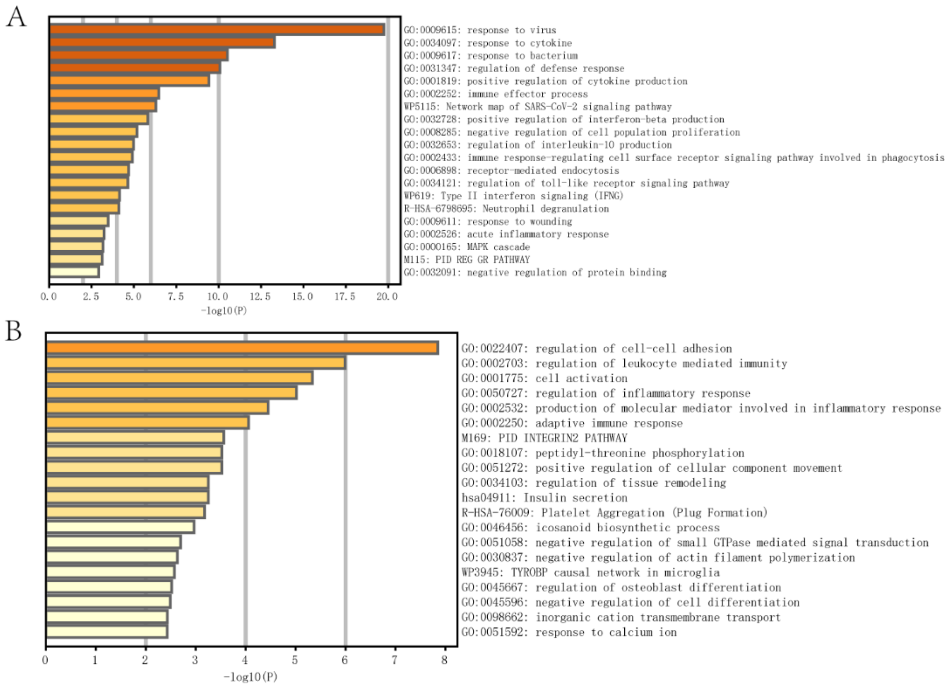

Supplementary Figure 3. Functional enrichment of up- and down-regulated genes based on Metascape database. (A) Volcano plot of up-regulated genes in EBV- and EBV+ NHLs groups. (B) Volcano plot of down-regulated genes in EBV- and EBV+ NHLs groups.

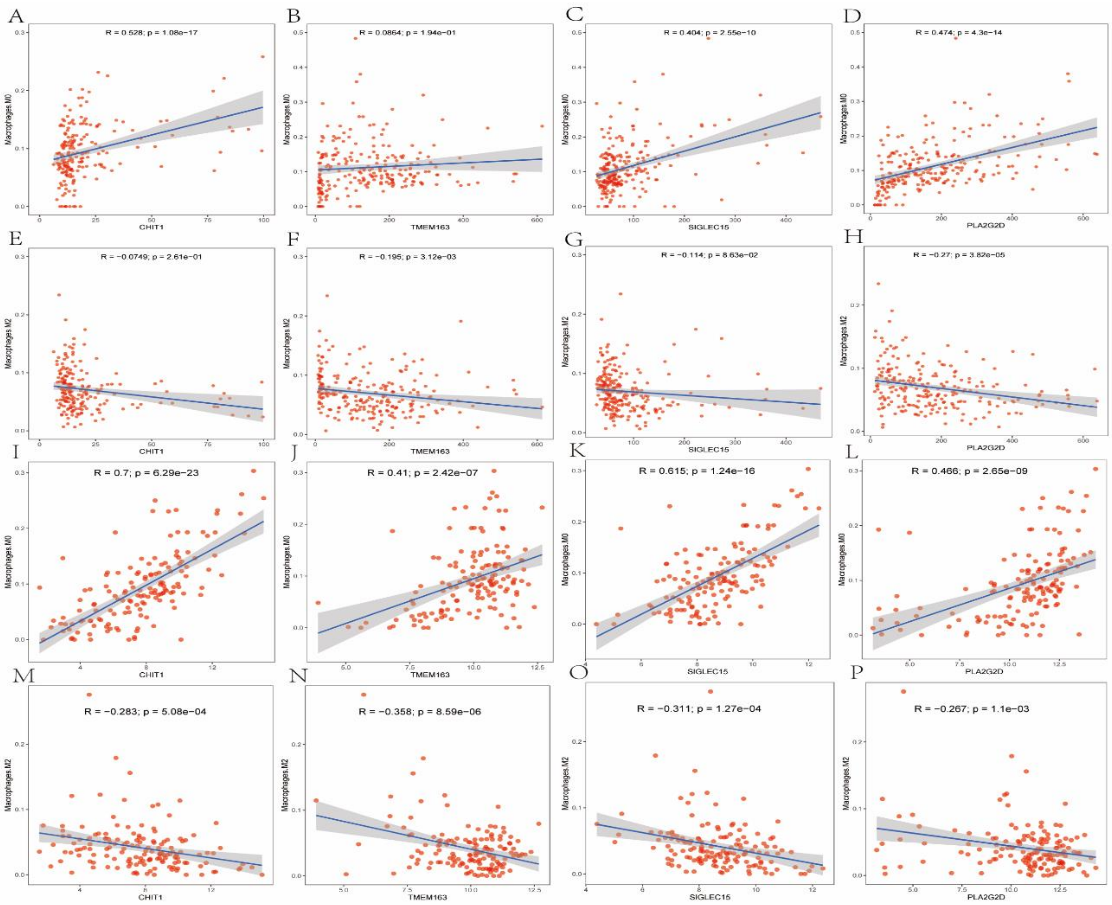

Supplementary Figure 4. (A-H) Correlation of four genes with infiltration of macrophage in NHLs without GC-DLBC (GSE132929). (I-P) Correlation of four genes with infiltration of macrophage in NHLs (GSE58445)

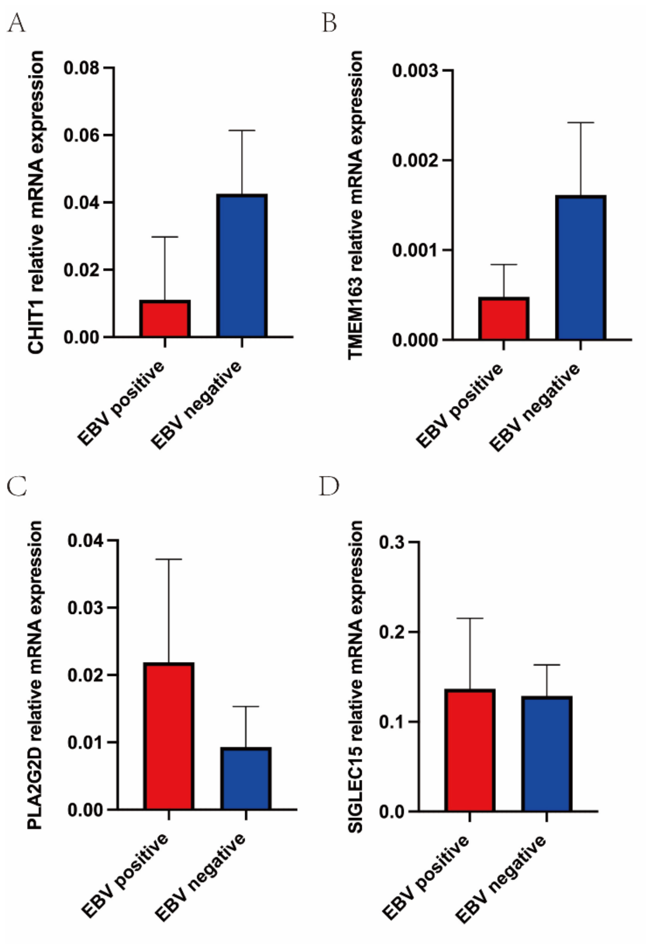

Supplementary Figure 5. RT-qPCR results of four hub genes in clinical samples (13 EBV+ and 13 EBV-).

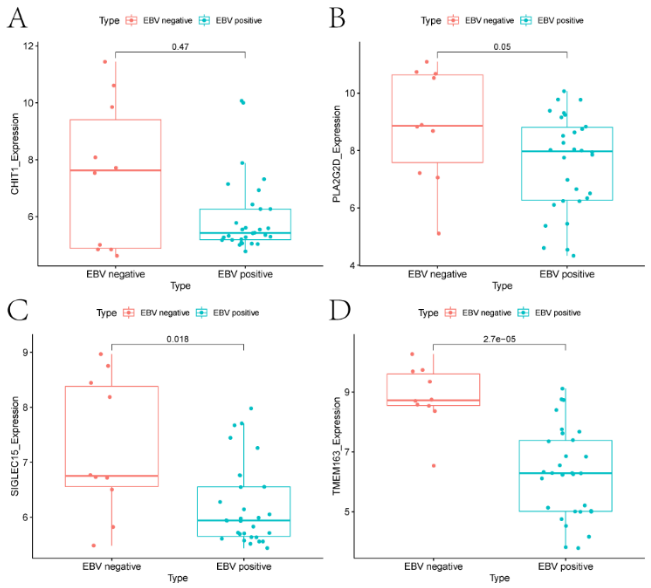

Supplementary Figure 6. The box plot shows the expression levels of four differential genes between two groups in immunocompromised NHLs from GSE38885 (30 EBV+ and 10 EBV-).

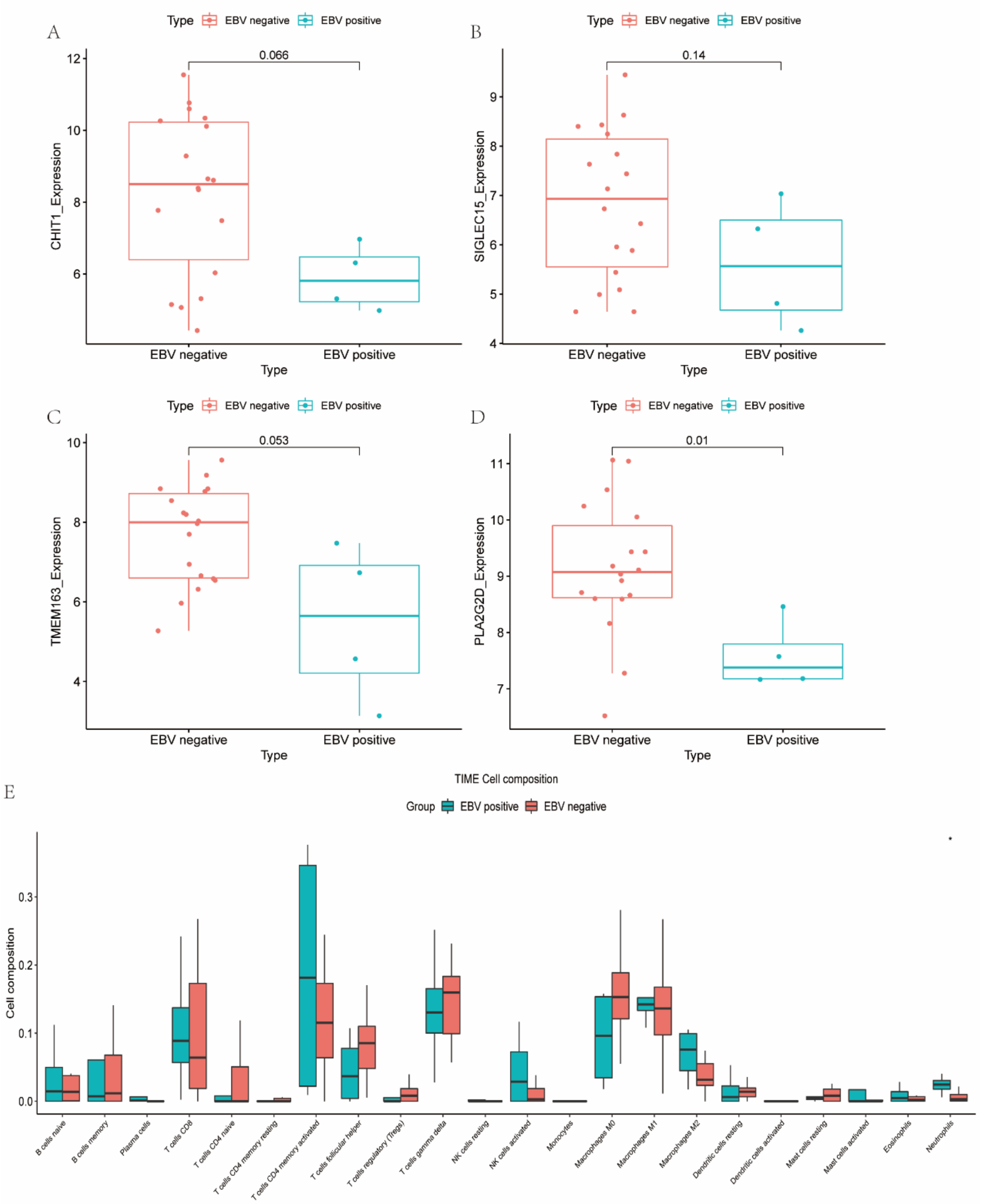

Supplementary Figure 7. (A-D) The box plot shows the expression levels of four differential genes between two groups in immunocompetent NHLs (GSE38885 and GSE34143), (4 EBV+ and 18 EBV-). (E) Infiltration levels of 22 immune cells in EBV+ and EBV- groups.

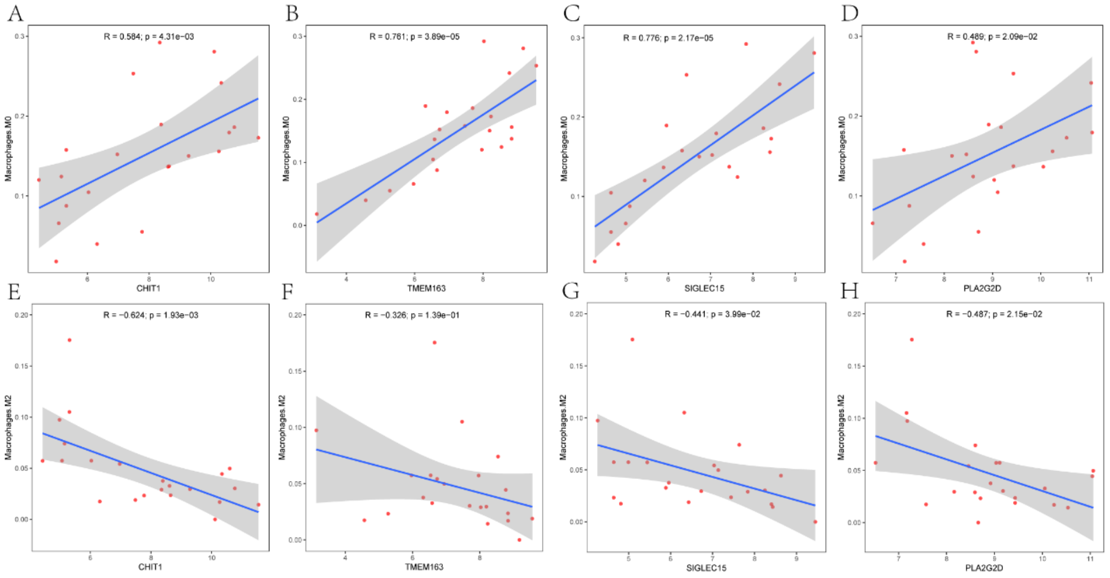

Supplementary Figure 8. Correlation of four genes with infiltration of macrophage in immunocompetent NHLs (GSE38885 and GSE34143).

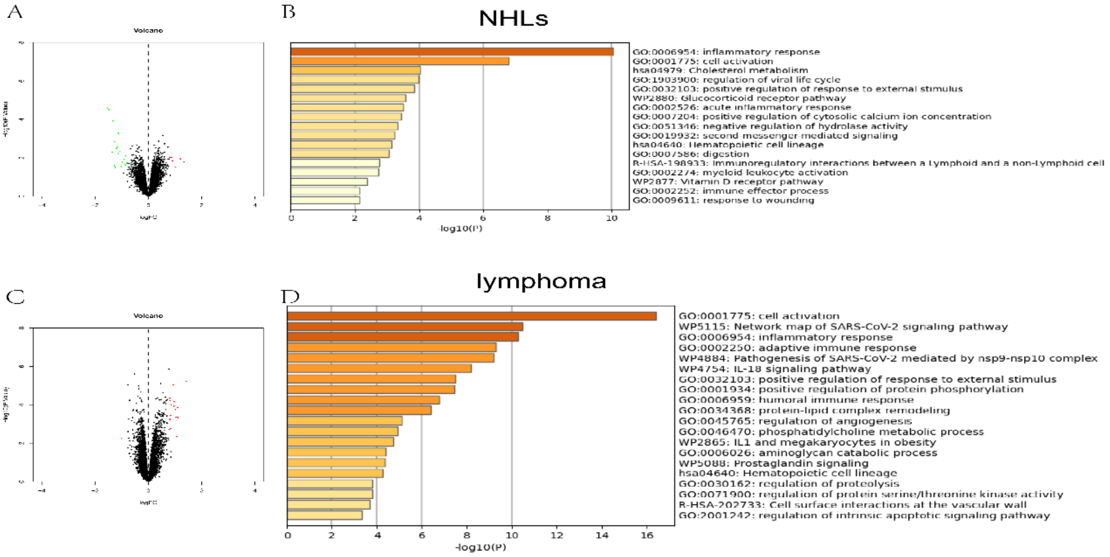

Supplementary Figure 9. (A) Volcano plot of differential genes in immunocompromised and immunocompetent NHL patients' groups (GSE38885, TCGA-DLBC and GSE58445), (40 immunocompromised and 220 immunocompetent). (B) Functional enrichment of differential genes in immunocompromised and immunocompetent NHL patients bases on Metascape database. (C) Volcano plot of differential genes in immunocompromised and immunocompetent lymphoma patients' groups (GSE38885 and GSE13996), (40 immunocompromised and 76 immunocompetent). (D) Functional enrichment of differential genes in immunocompromised and immunocompetent lymphoma patients bases on Metascape database.

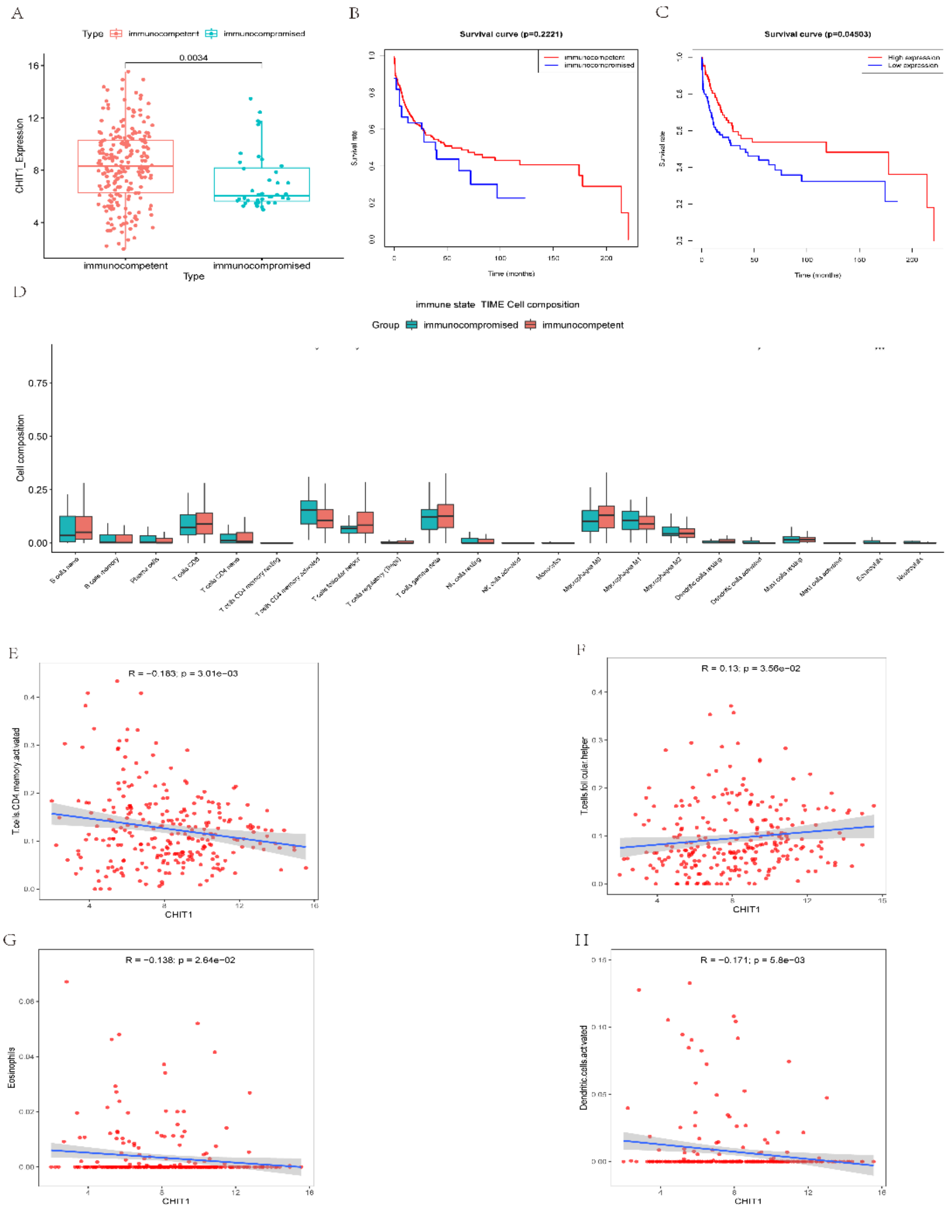

Supplementary Figure 10. (A) The box plot shows the expression level of CHIT1 between two groups in NHLs (GSE38885, TCGA-DLBC and GSE58445). (B) Survival analysis of lymphoma patients between immunocompromised and immunocompetent in NHLs. (C) Correlation of CHIT1 expression level with overall survival. (D) The box plot shows the differences of infiltration of 22 kinds of immune cells between two groups in NHLs. (E-H) Correlation of differential genes with infiltration of immune cells in NHL patients.

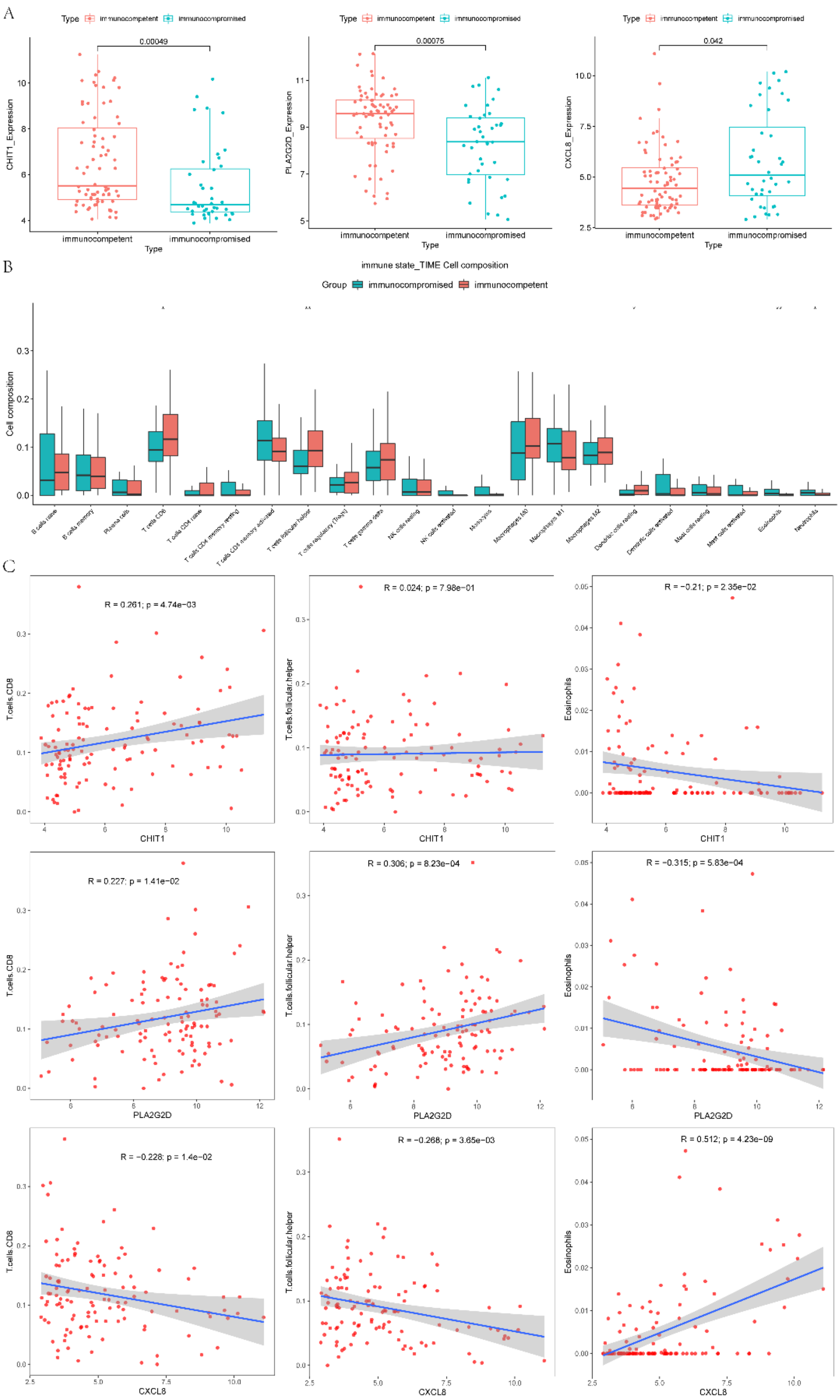

Supplementary Figure 11. (A) The box plot shows the expression level of three differential genes between two groups in lymphoma patients (GSE38885 and GSE13996). (B) The box plot shows the differences of infiltration of 22 kinds of immune cells between two groups in lymphoma patients. (C) Correlation of differential genes with infiltration of immune cells in lymphoma patients.

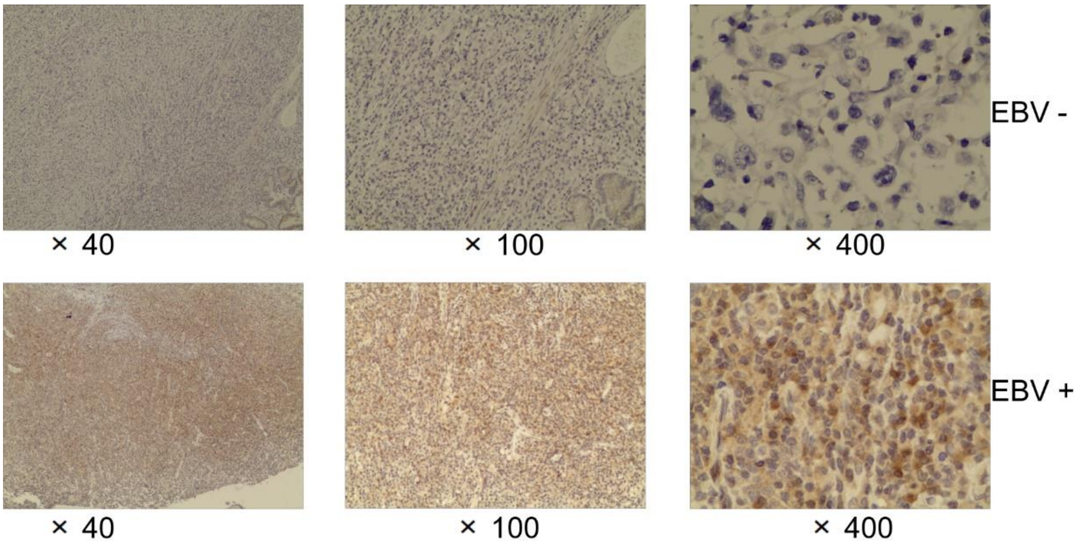

Supplementary Figure 12. Immunohistochemical results of PLA2G2D in EBV+ and EBV- groups

The immunohistochemical results did not agree with our bioinformatics analysis, but agree with the results of RT-qPCR.

2. Table explanation

The compound in Table 3

155518853

(2R,4S,5R,6R)-5-acetamido-2-[[(2R,3S,4S,5R,6S)-6-[(2R,3S,4R,5R,6R)-5-acetamido-6-(6-azidohexoxy)-4-hydroxy-2-(hydroxymethyl)oxan-3-yl]oxy-4-[(2S,4S,5R,6R)-5-acetamido-2-carboxy-4-hydroxy-6-[(1R,2R)-1,2,3-trihydroxypropyl]oxan-2-yl]oxy-3,5-dihydroxyoxan-2-yl]methoxy]-4-hydroxy-6-[(1R,2R)-1,2,3-trihydroxypropyl]oxane-2-carboxylic acid

155553698

(2R,4S,5R,6R)-5-acetamido-2-[[(2R,3S,4S,5R,6S)-4-[(2S,4S,5R,6R)-5-acetamido-2-carboxy-4-hydroxy-6-[(1R,2R)-1,2,3-trihydroxypropyl]oxan-2-yl]oxy-6-[(2R,3S,4R,5R,6R)-6-(6-azidohexoxy)-4,5-dihydroxy-2-(hydroxymethyl)oxan-3-yl]oxy-3,5-dihydroxyoxan-2-yl]methoxy]-4-hydroxy-6-[(1R,2R)-1,2,3-trihydroxypropyl]oxane-2-carboxylic acid

3. Supplementary tables

Table 1 The dataset corresponding to different case studies

Different case studies	Dataset
Differential gene analysis between EBV+ and EBV- lymphoma	GSE38885, GSE13996 (49 EBV+ vs 67 EBV-)
Differential gene analysis between EBV+ and EBV- NHLs	GSE38885 (31 EBV+ vs 34 EBV-)
Differential gene analysis between EBV+ and EBV- HLs	GSE13996 (18 EBV+ vs 33 EBV-)
Differential gene analysis between EBV+ and EBV- NHLs (only immunocompromised patients)	GSE38885 (30 EBV+ vs 10 EBV-)
Differential gene analysis between EBV+ and EBV- NHLs (only immunocompetent patients)	GSE38885, GSE34143 (4 EBV+ vs 18 EBV-)
Survival analysis between NHLs and HLs	GSE4475, GSE39133, GSE39134 (159 NHLs vs 58 HLs)
Single gene survival analysis	GSE4475, TCGA-DLBC (207 NHLs)
Immune infiltration analysis between EBV+ and EBV- lymphoma	GSE38885, GSE13996 (49 EBV+ vs 67 EBV-)
Immune infiltration analysis between EBV+ and EBV- NHLs	GSE38885 (31 EBV+ vs 34 EBV-)
Immune infiltration analysis between EBV+ and EBV- HLs	GSE13996 (18 EBV+ vs 33 EBV-)
Immune infiltration analysis between EBV+ and EBV- NHLs (only immunocompetent patients)	GSE38885, GSE34143 (4 EBV+ vs 18 EBV-)
Correlation between the differential genes and macrophage infiltration in NHLs	GSE38885 (65 NHLs), GSE132929 (227 NHLs), GSE58445 (147)
Correlation between the differential genes and macrophage infiltration in HLs	GSE13996 (51 NHLs)
Correlation between the differential genes and macrophage infiltration in immunocompromised NHLs	GSE38885 (40 immunocompromised NHLs)
Correlation between the differential genes and macrophage infiltration in immunocompetent NHLs	GSE38885, GSE34143 (22 immunocompetent NHLs)
total analysis according to different immune status in NHLs	GSE38885 (65 NHLs), GSE58445 (147 NHLs), TCGA-DLBC (48 NHLs); (40 immunocompromised vs 220 immunocompetent)
total analysis according to different immune status in lymphoma including HLs	GSE38885 (65 NHLs), GSE13996 (51 HLs); (40 immunocompromised vs 76 immunocompetent)

Table 2 Details of the datasets we used

datasets	immunocompetent	immunocompromised	EBV+	EBV-	diagnoses										
					DLBCL	BLL	BL	PTCL	aggressive B-NHL unclassifiable	atypical BL	FL	HGBL-NOS	MCL	MZL	HL
GSE38885	25	40	31	34	51	9	5	0	0	0	0	0	0	0	0
GSE13996	51	0	18	33	0	0	0	0	0	0	0	0	0	0	51
GSE34143	3	0	3	0	0	0	0	3	0	0	0	0	0	0	0
GSE4475	159	0	-	-	123	0	5	0	9	22	0	0	0	0	0
GSE39133	29	0	-	-	0	0	0	0	0	0	0	0	0	0	29
GSE39134	29	0	-	-	0	0	0	0	0	0	0	0	0	0	29
GSE132929	227	0	-	-	33	0	60	0	0	0	65	3	43	23	0
GSE58445	147	0	-	-	0	0	0	147	0	0	0	0	0	0	0
TCGA-DLBC	48	0	-	-	48	0	0	0	0	0	0	0	0	0	0
total	718	40	52	67	255	9	70	150	9	22	65	3	43	23	109

The meaning of the abbreviations in the table2: diffuse large B-cell lymphoma (DLBCL), burkitt-like lymphoma (BLL), burkitt lymphoma (BL), peripheral T cell lymphoma (PTCL), follicular lymphoma (FL), mantle cell lymphoma (MCL), medial zone lymphoma (MZL), hodgkin's lymphoma (HL)
