# Supplementary figures and images for: Identification and validation of an immunological microenvironment signature and prediction model for epstein-barr virus positive lymphoma: Implications for immunotherapy
Source: Front Oncol. 2022 Sep 29;12:970544. doi: 10.3389/fonc.2022.970544 (PMC9559214; doi:10.3389/fonc.2022.970544)

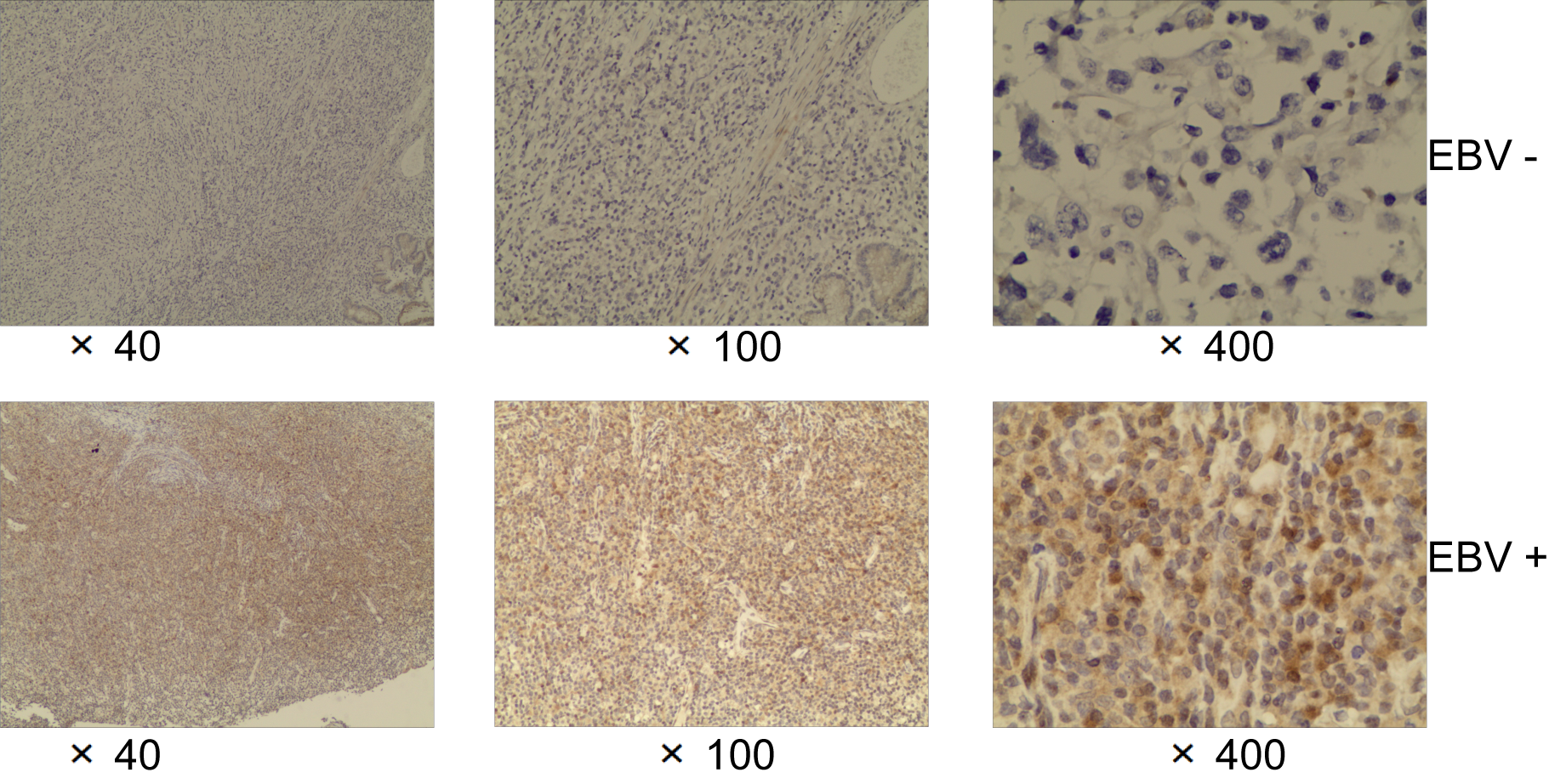

Supplement: Supplementary file 2 [file Image_1.tif]
